# Kindler Syndrome Presenting as Colitis in an Infant

**DOI:** 10.7759/cureus.43928

**Published:** 2023-08-22

**Authors:** Sara Idkaidak, Maram Albandak, Firas Alqarajeh, Osama N Dukmak, Jihad Imhaimeed, Nabil C. N. Khalil

**Affiliations:** 1 Pediatric Medicine, Al-Quds University, Jerusalem, PSE; 2 Internal Medicine, Al-Quds University, Jerusalem, PSE; 3 Pediatric Medicine, Palestine Red Crescent Society (PRCS) Hospital, Hebron, PSE; 4 College of Medicine, Al-Quds University, Jerusalem, PSE; 5 Internal Medicine, Al-Makassed Charitable Society Hospital, Jerusalem, PSE

**Keywords:** colitis, kindlin-1, failure to thrive, fermt1, kindler syndrome

## Abstract

Kindler syndrome (KS) is an autosomal recessive genodermatosis characterized by skin atrophy, blistering, photosensitivity, and mucosal inflammation. We present a unique case of KS with early and severe neonatal onset in a two-month-old female who presented with severe failure to thrive (FTT) and chronic diarrhea since birth. The infant also had multiple fluid-filled cysts on her foot since birth, which resolved and reappeared at different sites. Anemia, hyponatremia, and coloboma of the right iris were also observed. Whole exome sequencing revealed a homozygous mutation in the FERMT1 gene, confirming the diagnosis of KS. Our case demonstrates a distinct clinical phenotype involving severe colitis and FTT in addition to the typical skin manifestations of KS. This atypical presentation highlights the need for further investigations to gain insights into the impact of the kindlin-1 defect on organs beyond the skin and to explore potential therapeutic approaches for managing severe colitis in affected patients.

## Introduction

Kindler syndrome (KS) is an autosomal recessive genodermatosis, recently reclassified as a subtype of epidermolysis bullosa [1]. KS was first described in the 1950s by Theresa Kindler and is characterized by congenital blistering in acral regions, generalized progressive poikiloderma, mucosal inflammation, and varying degrees of photosensitivity [2]. Kindlin-1 is a protein primarily expressed in basal keratinocytes, the gut, and the kidney, and it is encoded by the FERMT1 gene. It serves as an intracellular focal adhesion protein that is crucial for the migration, proliferation, and adhesion of keratinocytes as well as the activation of integrins [3-5]. The highest expression of the FERMT1 gene can be found in keratinocytes, the colon, the kidney, and the placenta, while other organs have lower levels. This explains why KS primarily affects the skin and mucosal tissue. The severity of the disease varies from mild symptoms, such as skin ulcers and blisters, to more severe cases with extensive mucosal involvement, severe esophageal stenosis, anemia, and, rarely, colitis. Over 55 FERMT1 variants, including deletions, insertions, nonsense, splice-site, and missense alterations, have been documented in KS thus far in.

Herein, we describe an early and severe form of Kindler syndrome with a novel phenotype characterized by severe colitis, which has not been reported before. 

## Case presentation

**Figure 1:**
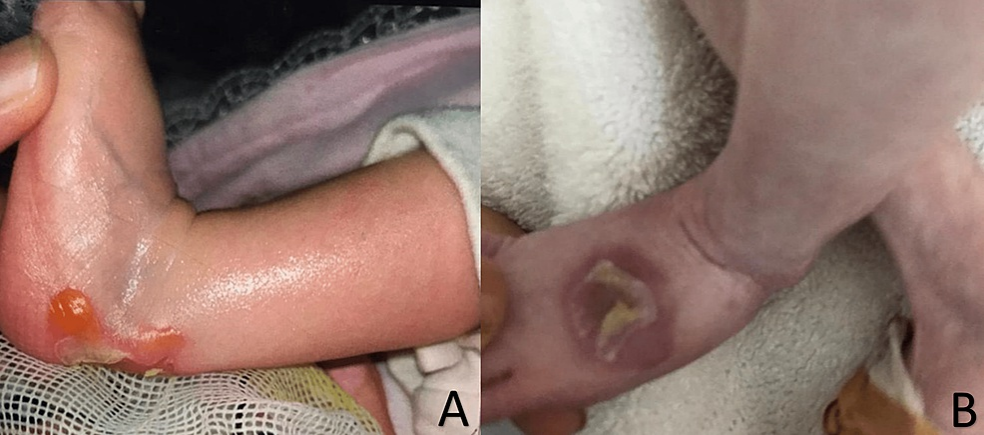
Multiple bilateral fluid-filled cysts on the lower limbs.

A two-month-old female infant was delivered full term by normal vaginal delivery with a birth weight of 3800 g and presented with severe failure to thrive (FTT) and chronic diarrhea, which had been persistent since birth. She has had multiple bilateral small fluid-filled cysts on the foot that usually resolve after some time and reappear at different sites since her birth (Figure 1). The lesions were seen by a dermatologist and were managed with moisture and fusidic acid creams.

Additionally, since the age of one week, she has had frequent, watery diarrhea in small amounts with no mucus or blood. At the age of two weeks, due to persistent diarrhea and significant weight loss (500 g), she was admitted to the neonatal intensive care unit (NICU) for 13 days. Throughout her hospitalization, she has had persistent hyponatremia, and a comprehensive laboratory workup revealed the results summarized in (Table 1).

**Table 1:** Laboratory values of the patient.

Parameters	Result	Normal Value
ALT	18 U/L	7-55 U/L
AST	25 U/L	8-48 U/L
Alkaline phosphatase	150 U/L	30-170 U/L
Total protein	3 g/dl	3-5 g/dl
Albumin	2.8 g/dl	2.5-4 g/dl
LDH	412 U/L	230-460 U/L
Cholesterol	95 g/dl	Up to 200 g/dl
Triglyceride	206 mg/dl	35-160 mg/dl
Hemoglobin (Hg)	6.8 g/dl	9.5-13 g/dl

At one month of age, the infant began experiencing non-projectile milky vomiting on approximately five occasions, resulting in her readmission for an eight-day period. Blood gas analysis revealed metabolic acidosis and hyponatremia. A stool examination for occult blood returned positive results. In an attempt to address these symptoms, her milk was switched to a soy-based formula, but without notable improvement. Consequently, she was discharged with an extensively hydrolyzed formula. While using this formula, the frequency of diarrhea decreased to four to five times per day, with a semisolid consistency.

At 45 days of age, the infant was readmitted due to dehydration and inadequate weight gain. Stool analysis detected the presence of *Entamoeba histolytica *cysts, leading to the administration of metronidazole for a duration of 10 days. Additionally, the sweat chloride test was negative, and 17 alpha-OH progesterone, aldosterone, and cortisol levels were normal. The infant remained hospitalized until two months of age when she was transferred to our hospital for further evaluation and management. 

Upon admission, the patient's weight was recorded at 2,510 g, which falls below the third percentile for her age. Physical examination revealed signs of cachexia, with loss of muscle mass. Additionally, ulcerative lesions were observed on the left foot and the middle finger of the left hand, exhibiting hyperemia and irregular borders. An eye examination further identified a coloboma of the right iris.

To determine the nature of diarrhea, the patient was kept on nothing-by-mouth (NPO) status for a period of 24 hours. During this fasting period, the frequency of diarrhea decreased but did not completely resolve. Consequently, the milk formula was switched to an amino acid-based formula (Neocate) at a volume of 40 cc every three hours (totaling 320 cc per day), providing 72 kcal/kg/day through a nasogastric tube (NGT). The new formula resulted in a decrease in bowel movements to five times per day, with a semisolid consistency. Over time, feeding was gradually increased to 60 cc every three hours (totaling 480 cc per day), providing 107 kcal/kg/day. Total parenteral nutrition (TPN) was administered for only three days on admission and was then discontinued following the removal of the central line. Over the course of one month, the patient experienced a weight gain of 800 grams.

Various investigations were conducted to assess the patient's condition, including serum alpha-one antitrypsin levels, urine analysis for reducing substances, abdominal ultrasound, coagulation profile, stool analysis, triglyceride levels, liver enzymes, cholesterol levels, and zinc levels. All of these tests returned normal results. However, the complete blood count (CBC) revealed anemia without neutropenia or thrombocytopenia. The patient's hemoglobin level was 6.8; as a result, a packed red blood cell (PRBC) transfusion was administered. Notably, the patient’s sodium level was 127 mEq/L. To address this, the formula milk was prepared using 0.18 normal saline to increase the sodium content, successfully resolving the issue.

**Figure 2:**
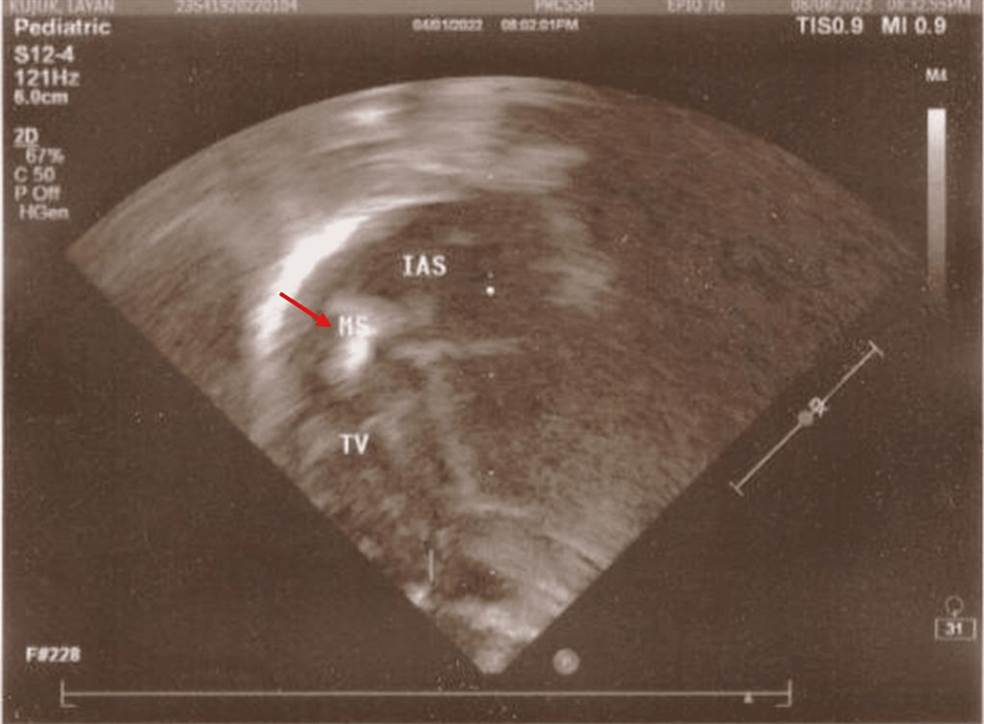
Echocardiogram depicting a substantial dynamic mass measuring 10x3 mm (red arrow) within the right atrium. MS, mass; IAS, interatrial septum; TV, tricuspid valve.

An echocardiogram revealed the presence of a large fluctuating mass measuring 10x3 mm in the right atrium (Figure 2). To rule out infective endocarditis, three blood cultures were obtained, but all yielded negative results. Whole exome sequencing was performed using the IDT xGen Exome Research Panel v2.0 (IDT, IA, USA) for protein-coding regions and the xGen Human mtDNA Research Panel v1.0 for mitochondrial DNA. Utilizing the Illumina NOVASEQ 6000 platform, we conducted paired-end sequencing with 100-base reads, ensuring optimal coverage and depth. This method adhered to stringent European Molecular Genetics Quality Network (EMQN) standards. Variants were carefully filtered, excluding intronic variants >8 base pairs from splice sites, synonymous variants >3 base pairs from splice sites, and variants with >1% minor allele frequencies in the GnomAD database. It uncovered a homozygous mutation in the FERMT1 gene (c.137_140del) confirming the diagnosis.

Ultimately, the patient was discharged with instructions to follow an amino acid-based formula. Additionally, a plan was put in place to perform a colonoscopy if the patient reexperienced diarrhea or bloody stool. Treatment for colitis with 5-aminosalicylic acid was considered if the patient's condition did not improve. In a follow-up after two weeks, the patient had good weight gain with decreased frequency of diarrhea.

## Discussion

KS is an uncommon autosomal recessive genodermatosis characterized by skin atrophy, blistering, photosensitivity, hyper or hypopigmentation, heightened sensitivity to light, and an increased risk of developing aggressive squamous cell carcinoma [6]. Approximately 400 afflicted individuals have been recorded globally since the first description of KS in 1954. People of any ethnicity can be affected, and there is no gender preference. Typically, patients with KS begin showing symptoms during infancy, and the condition progresses with age [7]. Some individuals with mild symptoms are not diagnosed until later in life. The condition often presents at birth as trauma-induced skin blistering, which is more pronounced on the limbs, and it tends to recede with maturity, becoming rare in adulthood [8].

Kindlin-1, a small GTPase of the Rho family, functions as a connecting link of the actin cytoskeleton and a modulator of cell adhesion and polarity. When mutations occur in the corresponding gene (KIND1), KS occurs. The syndrome manifests in various ways, such as skin blistering, poikiloderma, photosensitivity, and an increased risk of developing cancer [9]. Some individuals with Kindler syndrome also experience gastrointestinal symptoms, although it is not yet clear whether these symptoms directly indicate the presence of the syndrome or are merely coincidental [10]. What makes our case particularly unique is the combination of its severity at such an early age and the prominence of gastrointestinal complaints as the primary presenting issue.

Common mucosal symptoms of KS include hemorrhagic mucositis and gingivitis, periodontal disease, early tooth loss, and labial leukokeratosis. In addition, ectropion, urethral stenosis, and severe phimosis are other common mucosal abnormalities. Periodontitis, mucosal strictures, and aggressive squamous cell carcinomas are among the worst long-term consequences of KS [8].

Kindlin-1 is expressed in gastrointestinal epithelial cells, and its loss results in epithelial barrier injury and dysfunction, which explains the inflammatory intestinal phenotype of Kindler syndrome. Kindlin-1's presence is essential for maintaining the integrity of gastrointestinal tissues in individuals with Kindler syndrome [11]. In a previous study that examined the distribution and role of kindlin-1, researchers utilized indirect immunofluorescence (IIF) to investigate its presence in various tissues, including human oral mucosa, small intestine, colon, and rectum. The findings revealed that, similar to the skin, kindlin-1 displayed polarization in the basal cells positioned at the oral mucosa's basement membrane [12]. While kindlin-1 was shown to be localized in the plasma membrane of the epithelial cells of the colon and rectum, it was nearly impossible to detect Kindlin-1 in the terminal ileum [13].

Furthermore, it is noteworthy that the KIND1 gene, responsible for encoding the short kindlin-1 isoform, exhibits mutations within exons two to seven in all cases involving intestinal manifestations. These mutations result in a wide spectrum of outcomes. Specifically, a profound phenotype has been associated with a complete absence of the protein, often observed with the p.E304X mutation. Conversely, a milder form of the condition has been linked to the expression of truncated kindlin-1, often seen in conjunction with the p. Q226fsX16 mutation [14]. Interestingly, certain patients present at a young age with severe gastrointestinal features, emphasizing the influence of specific mutation types (missense, nonsense, exon or intron location, splicer) on disease severity [15].

Fuchs-Telem et al. documented the cases of two patients who exhibited a homozygous deletion of four base pairs (c.137-140delTAGT) within the second exon of FERMT1. This specific mutation resulted in a premature termination of protein translation (p. Leu46X), ultimately leading to the development of Kindler syndrome. Notably, the reported patients predominantly presented with skin manifestations [16]. In our patient, however, the same mutation was observed, but the clinical manifestations were notably more severe, extending beyond skin involvement [14].

The neonatal onset of Kindler syndrome is a relatively uncommon occurrence; however, our case highlights its existence. Early detection of this condition bears crucial clinical implications as it allows for better prognostication of potential future complications, appropriate healthcare management, and comprehensive genetic counseling for affected individuals and their parents [17]. To this end, genetic studies, such as exome sequencing analysis utilizing DNA samples, play a pivotal role in facilitating accurate diagnosis and providing valuable insights into the condition's underlying genetic basis and thus prognosis.

## Conclusions

Our findings document a unique and infrequent neonatal presentation of Kindler syndrome in a two-month-old patient. This presentation appears to represent a novel phenotype characterized by severe colitis and failure to thrive, distinct from the more typical skin manifestations. This warrants further investigations to gain insights into the impact of kindlin-1 defect on organs beyond the skin and to explore potential therapeutic approaches for managing severe colitis in affected patients.
